# Early intestinal microbiota changes in aged and adult mice with sepsis

**DOI:** 10.3389/fcimb.2022.1061444

**Published:** 2022-12-27

**Authors:** Yangyang Yuan, Shaohua Liu, Xianfei Ding, Ying Li, Xiaojuan Zhang, Heng Song, Xueyan Qi, Zihao Zhang, Kaiyuan Guo, Tongwen Sun

**Affiliations:** ^1^ General Intensive Care Unit, The First Affiliated Hospital of Zhengzhou University, Henan Key Laboratory of Critical Care Medicine, Henan Engineering Research Center for Critical Care Medicine, Zhengzhou, China; ^2^ Zhengzhou Key Laboratory of Sepsis, Zhengzhou, China; ^3^ Academy of Medical Sciences, Zhengzhou University, Zhengzhou, China; ^4^ Sanquan College Of Xinxiang Medical University, Xinxiang, China

**Keywords:** sepsis, intestinal microbiota, aged mice, adult mice, 16S

## Abstract

**Background:**

The mortality rate associated with sepsis in elderly individuals is higher than that in younger individuals. The intestinal microbiota has been demonstrated to play an important role in the occurrence and development of sepsis. The purpose of this study was to investigate the differences in the intestinal microbiota between aged and adult mice with sepsis.

**Methods:**

Thirty male C57BL mice were randomly divided into two groups: 15 in the adult group (AD group) and 15 in the age group (Age group). All the mice underwent caecal ligation and puncture to induce sepsis. Mice faeces were collected, and analysed using 16S rRNA sequencing. The liver and colon tissues were collected.

**Results:**

There were significant differences in intestinal microbiota composition between the two groups. Compared with adult sepsis mice, the diversity of intestinal microbiota in the aged group was significantly reduced and the structure of dominant intestinal microbiota was changed. In the Age group, the microbiota associated with inflammatory factors increased, and the microbiota associated with the production of SCFAs (Ruminiclostridium, Prevotellaceae_UCG-001, Rikenella, Parabacteroides, Oscillibacter, Odoribacter, Muribaculum, Lachnoclostridium, Intestinimonas, Faecalibaculum, Anaerotruncus, Alloprevotella and Absiella) decreased. The metabolic pathways related to the microbiota also changed. Moreover, the proportion of inflammatory factors in Age group was higher than that in AD group.

**Conclusion:**

Our results showed that there were significant differences in the abundance and structure of microbiota between aged and adult sepsis mice, Aged sepsis mice have more severe intestinal microbiota destruction and liver tissue inflammation than adult sepsis mice.

## Introduction

Sepsis is a life-threatening organ dysfunction caused by the maladjusted response of the host to infection (Shankar-Hari et al., 2016). Failure of regulatory mechanism during sepsis may lead to uncontrolled inflammation, and excessive activation of inflammatory reaction may lead to organ damage (Rittirsch et al., 2008). Sepsis is associated with a high mortality rate, approximately one-quarter of the global mortality rate (Vincent et al., 2014; Haak and Wiersinga, 2017). Sepsis is regarded as a “typical disease of the elderly” (Brakenridge et al., 2019). Age is an independent predictor of mortality in severely sick patients with sepsis, especially those over the age of 65, and sepsis morbidity and in-hospital mortality rates have increased exponentially (Milbrandt et al., 2010). Therefore, studying the difference between elderly patients and young patients with sepsis and identifying how the mechanism of sepsis differs in elderly patients is a problem to be solved.

Destruction of intestinal microbiome predisposes to sepsis, and has a negative impact on the results of sepsis (Fay et al., 2017; Agudelo-Ochoa et al., 2020; Sun et al., 2022). There are abundant bacteria in the intestinal microbiota of healthy people. The Bacteroidetes and Firmicutes are the most abundant, accounting for more than 90% of the intestinal microbiota (Adak and Khan, 2019). It is generally believed that the ratio of Firmicutes to Bacteroidetes is strongly correlated with health and disease (Mariat et al., 2009). Bacteroidetes and Firmicutes are major producers of short chain fatty acids (SCFAs), which can promote the antibacterial activity of macrophages and regulate the immune function of T cells (Smith et al., 2013; Schulthess et al., 2019; Miller et al., 2021). Butyric acid plays an important role in maintaining the integrity of the colon epithelium, and butyric acid is the main raw material for the utilization of the colon epithelium (Nava et al., 2011; Leonel and Alvarez-Leite, 2012; Morrison and Preston, 2016; Tanca et al., 2017). Propionate and its receptor GPR41 regulate Ang II levels and myocardial I/R injury (Deng et al., 2022). The intestinal microbiota and their metabolites are closely related to immunity, endocrinology and inflammation (Belkaid and Hand, 2014; Lin and Zhang, 2017; Deng et al., 2022). The imbalance in gut microbiota will cause a series of diseases, such as obesity, fatty liver and inflammatory diseases (Turnbaugh et al., 2006; Frank et al., 2007; Liu et al., 2018). The intestinal microbiota is an important part of the intestinal barrier. The breakdown of intestinal barrier function and microbiota translocation hasten the progression of sepsis or lead to sepsis (Sun et al., 2022). Patients with sepsis have less fecal SCFAs, which may worsen intestinal epithelial integrity and immune dysfunction in sepsis (Shimizu et al., 2006; Hotchkiss et al., 2013; Yamada et al., 2015). Furthermore, some studies have shown that patients with decreased microbial diversity and increased abundance of pathogenic bacteria are more susceptible to sepsis (Shimizu et al., 2006; Hotchkiss et al., 2013; Yamada et al., 2015; Adhi et al., 2019; Adelman et al., 2020). In this study, we studied the differences in intestinal microbiota between adult and aged mice with sepsis.

## Materials and methods

### Animal experiments

Thirty male C57BL mice (6–8 weeks) obtained from Beijing Vital River Laboratory Animal Technology (Beijing, China) were kept in a specific-pathogen-free (SPF) animal laboratory with unlimited access to food and water in a temperature-controlled and light-regulated environment (20-25°C, 1:1 light dark cycle). The mice were randomly categorized into two groups: the adult (AD) group (n = 15) and the aged group (n = 15). The mice in the aged group were fed until 20-21 months. Sepsis was induced in both groups by caecal ligation and puncture (CLP). The establishment of CLP model was based on our previous research (Cui et al., 2020; Liang et al., 2022). The mice were anaesthetized with an intraperitoneal dose of pentobarbital (30 mg/kg). Half of the caecum was ligated, and the caecum was punctured twice with a No. 21 needle. The contents of the caecum were extruded, treated and put back, and two layers of sutures were used to close the incision (muscle layer and skin). In addition, normal saline (37°C; 1 ml/100 g) was injected subcutaneously into the mice. The mice were rewarmed for 1 h and then returned to the cage. The mice were watered, fed and maintained on a light/dark (12/12 hours) cycle. At 24 hours after CLP treatment, 5 mice in the aged group and 8 mice in the AD group were alive. We chose to collect the feces of surviving rats of the two groups, 24 hours after operation. All the experiments in this study were approved by the Life Science Ethics Review Committee of Zhengzhou University.

### 16s rRNA gene sequencing

16s rRNA gene sequencing is a high-throughput sequencing method for all bacteria in specific environmental (or specific habitat) samples and is used to study the composition of microbial populations in those samples, interpret the diversity, richness and population structure of microbial populations, and to explore the relationship between microorganisms and the environment or host. The faeces from both groups of mice were collected and kept at -80°C. DNA was extracted from various samples using the E.Z.N.A. ^®^Stool DNA Kit (D4015, Omega, Inc., USA). Total DNA was eluted in 50 L of elution buffer and stored at -80°C until PCR assessment. To eliminate the possibility of false-positive PCR results from the negative control, ultrapure water, instead of sample solution, was used throughout the DNA extraction process. AMPure XT beads (Beckman Coulter Genomics, Danvers, MA, USA) were used to purify the PCR products, and Qubit was used to quantify them (Invitrogen, USA). The amplicon pools were prepared for sequencing, and the size and quantity of the amplicon library were determined using an Agilent 2100 Bioanalyzer (Agilent, USA) and the Library Quantification Kit for Illumina (Kapa Biosciences, Woburn, MA, USA). The libraries were sequenced on the NovaSeq PE250 platform. After computer sequencing, the dual terminated data were patched by overlap, and a quality check and chimaera filtering were conducted to obtain high-quality clean data. The Divisive Amplicon Denoising Algorithm 2 (DADA2) was used to obtain representative sequences with single base accuracy, calculated using the QIIME2 microbiome bioinformatics platform. We used the SILVA (Release 132, https://www.arb-silva.de/documentation/release-132/) and NT-16S databases for species classification and subsequent analysis.

### Quantification of mRNAs by RT–PCR

We extracted total RNA from liver tissue using TRIzol reagent (Takara, Tokyo, Japan) and determined the RNA concentration and purity by ultraviolet spectrophotometry. A TaqMan reverse transcription kit (UE, Suzhou, China) was used to reverse transcribed mRNA and synthesize corresponding cDNA. Gene expression was normalized using reduced glyceraldehyde-phosphate dehydrogenase (GAPDH) expression. The gene primers were *TNF-α* forward primer, CCACCACGCTCTTCTGTCTAC, reverse primer, AGGGTCTGGGCCATAGAACT;

IL-6 forward primer, TGATGCACTTGCAGAAAACA, reverse primer, ACCAGAGGAAATTTTCAATAGGC; ccl2 forward primer, CCTGCTGTTCACAGTTGCC, reverse primer, ATTGGGATCATCTTGCTGGT; ccl3 forward primer, ACCATGACACTCTGCAACCA, reverse primer, GTGGAATCTTCCGGCTGTAG; ccl4 forward primer, CATGAAGCTCTGCGTGTCTG, reverse primer, GAAACAGCAGGAAGTGGGAG; ccl5 forward primer, CCACTTCTTCTCTGGGTTGG, reverse primer, GTGCCCACGTCAAGGAGTAT; ccl7 forward primer, CTGCTTTCAGCATCCAAGTG, reverse primer, TTGCCTCTTGGGGATCTTTTG; ccl8 forward primer, TCTTTGCCTGCTGCTCATAG, reverse primer, GAAGGGGGATCTTCAGCTTT; CXCL1 forward primer, ACCCAAACCGAAGTCATAGC, reverse primer, TCTCCGTTACTTGGGGACAC; CXCL10 forward primer, CTCATCCTGCTGGGTCTGAG, reverse primer, CCTATGGCCCTCATTCTCAC; and GAPDH forward primer, TGACCTCAACTACATGGTCTACA, reverse primer, CTTCCCATTCTCGGCCTTG.

### Statistical analysis

Species difference tests were performed using Fisher’s exact test, the Mann–Whitney U test, or the Kruskal–Wallis test. We defined P < 0.05 as significantly different species. Alpha and beta diversity indexes were calculated using QIIME2 and plotted using the R package. Linear discriminant analysis (LDA) effect size (LEfSe) was carried out; the purpose of which is to compare two or more groups and uncover species with significant abundance differences. All distinctive species were detected using the Kruskal–Wallis rank sum test. To acquire significantly distinct species, the difference in species abundance between various groups was detected. The Wilcoxon rank sum test was used to determine whether all subspecies of the significantly distinct species from the previous stage converged to the same categorization level. Finally, LDA was used to obtain the final differential species (biomarker). PICRUSt2 was used for functional prediction (https://github.com/picrust/picrust2). According to the t test, the threshold value of P value < 0.05 was considered a significant difference. The results showed that there were statistically significant differences in the abundance data in the functional database (confidence interval 95%). Statistical analysis was performed using R-package and GraphPad Prism version 5.0 (GraphPad Software, La Jolla, CA, USA).T-tests and Mann-Whitney test were used for two-group comparisons. P < 0.05 was considered statistically significant.

## Results

### Mortality of mice with sepsis

To explore the difference in mortality between aged mice and adult mice, we recorded the mortality of the two groups 24 hours after modelling. In this experiment, we raised thirty mice and generated a CLP model. After 24 hours of modelling, 8 mice survived in the AD group, and 5 mice survived in the Age group. The 24-hour mortality rates of the two groups were 47% and 67%, respectively(**Supplement Table 1**).

### Changes in inflammatory factors

**Figure 1:**
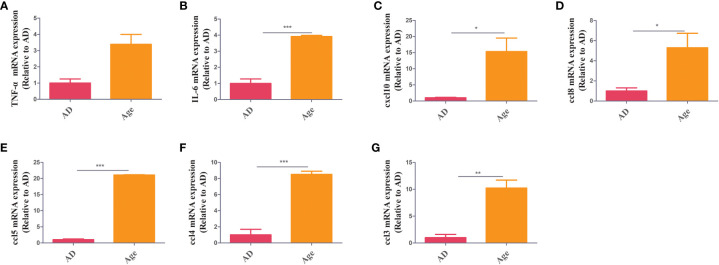
Inflammatory factors. RT–PCR showed that in colon tissue, the Age group exhibited increased levels of inflammatory factors (*TNF-α, IL-6, ccl3, ccl5, ccl8, cxcl10 and ccl4*) when compared with the AD group *P< 0.05, **P< 0.01, ***P< 0.001.

To study the changes of inflammatory factor levels between two groups, The mRNA expression of inflammatory factors in liver and colon tissue were measured. In colon tissue, the mRNA expression of inflammatory factors in the aged mice were higher than those in the adult mice. The levels of *TNF-α, IL-6, ccl3, ccl5, ccl8, CXCL10,* and *ccl4* increased in Age group (**Figure 1**). We also analysed inflammatory factors in liver tissue. The mRNA expression of inflammatory factors in the age group was higher (**Supplementary Figure 1**). The results showed that the Age group had higher levels of inflammatory factors, which may be associated with the poor prognosis and high mortality rate in elderly patients with sepsis.

### Differences in microbiota diversity

To study the differences in diversity of the intestinal microbiota between the two groups, we first used a Venn diagram to visually describe microbiota abundance differences between two groups. According to obtained eigenvalue abundance table, the number of common features of each group was calculated, and the number of common and unique features of each group was intuitively presented through a Venn diagram (**Supplement Figure 2A**). The abundance curve was also plotted. It assess the richness and variety from each sample, the number of features in various samples were compared at the same sequencing depth. If the curve steeply increases, the amount of sequencing data is insufficient. If the curve is flat, the amount of sequencing data has reached its limit. The Shannon curve was flat, indicating that the amount of sample sequencing data was sufficient (**Supplement Figure 2B**). The rank abundance curve was used to explain both the abundance and uniformity of species in a sample. The results showed that the abundance and uniformity of species were reliable (**Supplement Figure 2C**).

**Figure 2:**
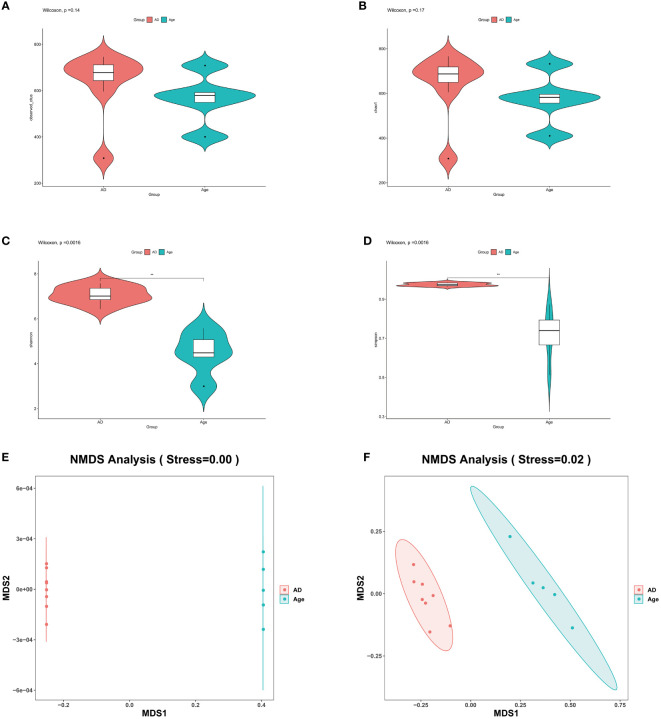
Microbial diversity. **(A–D)** The main purpose of alpha diversity is to reflect species richness, evenness, and sequencing depth. To reflect richness and uniformity, we used the Chao1, observed species, Shannon, and Simpson indexes. **(E, F)** β diversity reflects the microbial richness within and between groups. We used NMDS to observe the differences between samples. As shown in the figure, the two samples are far apart. The two groups of microbiota differ significantly, and the difference is statistically significant. *P< 0.05, **P< 0.01, ***P< 0.001.

The similarity of operational taxonomic units (OTUs) was 97%. Each OTU is usually considered a microbial specie. The two groups of observed OTUs were compared and analysed by using the Wilcoxon method. The results showed that compared to the AD group, the Age group showed a decreasing trend but the difference was not statistically significant (**Figure 2A**). Alpha diversity is used to reflect species richness, homogeneity, and sequencing depth within a certain area or ecosystem. The species diversity between distinct environmental communities is referred to as beta diversity. Beta diversity and alpha diversity describe an environmental community’s overall variety or biological heterogeneity. We analysed alpha diversity, and the findings revealed that, when compared to the AD group (**Figures 2B–D**), the diversity of the microbiota in the Age group showed a decreasing trend and the Shannon and Simpson index were statistically significant. However, chao1 index was not statistically significant. (Shannon P=0.0016, Simpson P=0.0016, Chao1P=0.17). We used Non-metric multidimensional scaling (NMDS) for beta diversity, unweighted unifrac and weighted unifrac were used to evaluate the analysis. (**Figures 2E, F**). There were considerable microbial differences between two groups and structure of the intestinal microorganisms in the two groups were significantly different.

### Differences in abundance of the dominant microbiota

**Figure 3:**
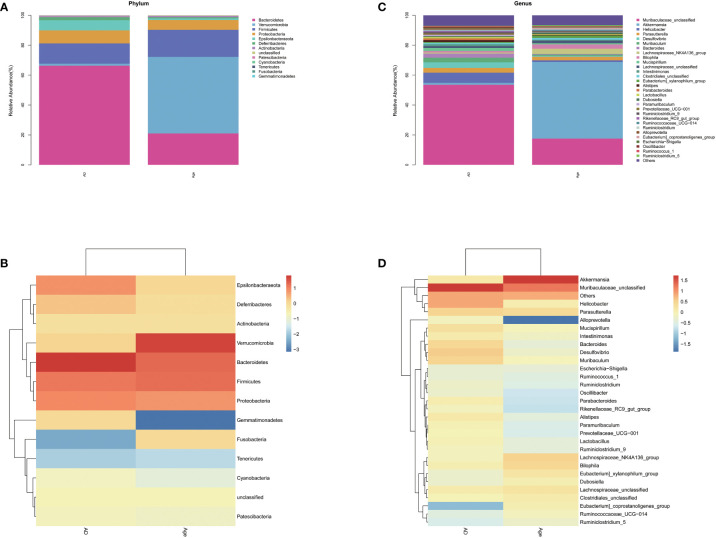
Relative abundance. Relative microbiota distribution between the two groups. **(A, B)** phylum level. **(C, D)** genus level.

We analysed the microbiota structure at the phylum and genus levels to evaluate the differences in the microbiota structure between the adult mice and the aged mice. The results showed that the microbiota structure of the adult mice was significantly different from that of the aged mice. At the phylum level (**Figures 3A, B**), *Bacteroidetes, verrucomicrobia, Firmicutes* and *Proteobacteria* account for the main part. Compared with AD group, *Bacteroides* in the Age group decreased and *verrucomicrobia* increased (**Supplement Figure 3**). At the Genus level (**Figures 3C, D**), the microbiota associated with inflammation, such as *Robinsoniella, Eubacterium Coriobacteriaceae-Ucg-002, Clostridioides, Akkermansia* and other microbiota increased significantly when compared with the AD group, while the microbiota related to the production of short chain fatty acids(SCFAs), such as *Ruminiclostridium, Rikenella, Prevotellaceae Ucg-001, Parabacteroides, Oscillibacter, Odoribacter, Muribaculum, Lachnoclostridium, Intestinimonas, Faecalibaculum, Anaerotruncus, Alloprevotella* and *Absiella* decreased significantly. We performed relative abundance analysis in **Supplementary Figure 3**. The findings suggested substantial differences in dominant microbiota between two groups. In addition, we analyzed the correlation between intestinal microbiota and inflammatory factors. In liver and colon tissue, the correlation analysis between inflammatory factors and microbiota showed that all inflammatory factors were positively correlated with Verrucomicrobia, Robinsonella, Firmicutes, Eubacterium, Coriobateriaceae_ UCG-002, Clostridioides and Akkermania (**Supplementary Figure 4**)

**Figure 4:**
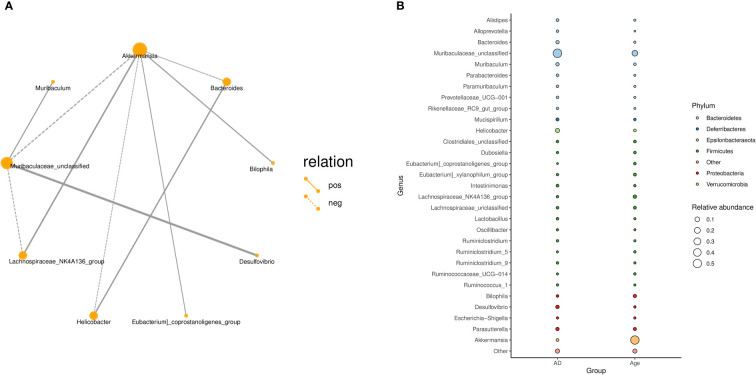
**(A)** Correlation network diagram: Different nodes in the network diagram represent different dominant genera. The connection between nodes indicates that there is a correlation between the two genera. By default, we show the relationship pair of correlation coefficientsRho> 0.4. **(B)** Bubble plot: The species annotation information and relative abundance (circle size) at the genus level in various sample groups as well as the species annotation information (circle colour) of the species-corresponding gate.

In addition, we explored the correlation between the microbiota and calculated the correlation between species through the abundance and changes in the different species in each sample. The results showed that Akkermansia was positively correlated with the Lachnospiraceae-NK4A136-group, Bilophil group, and Eubacterium coprostanoligenes group and negatively correlated with Bacteroides, Helicobacter, and Muribaculaceae_ Unclassified (**Figure 4A**). **Figure 4B** describes the taxonomic information of the two groups, and the results showed that the Age group was significantly correlated with the *Akkermansia*, while the AD group was significantly correlated with the *Muribaculaceae*-Unclassified.

**Figure 5:**
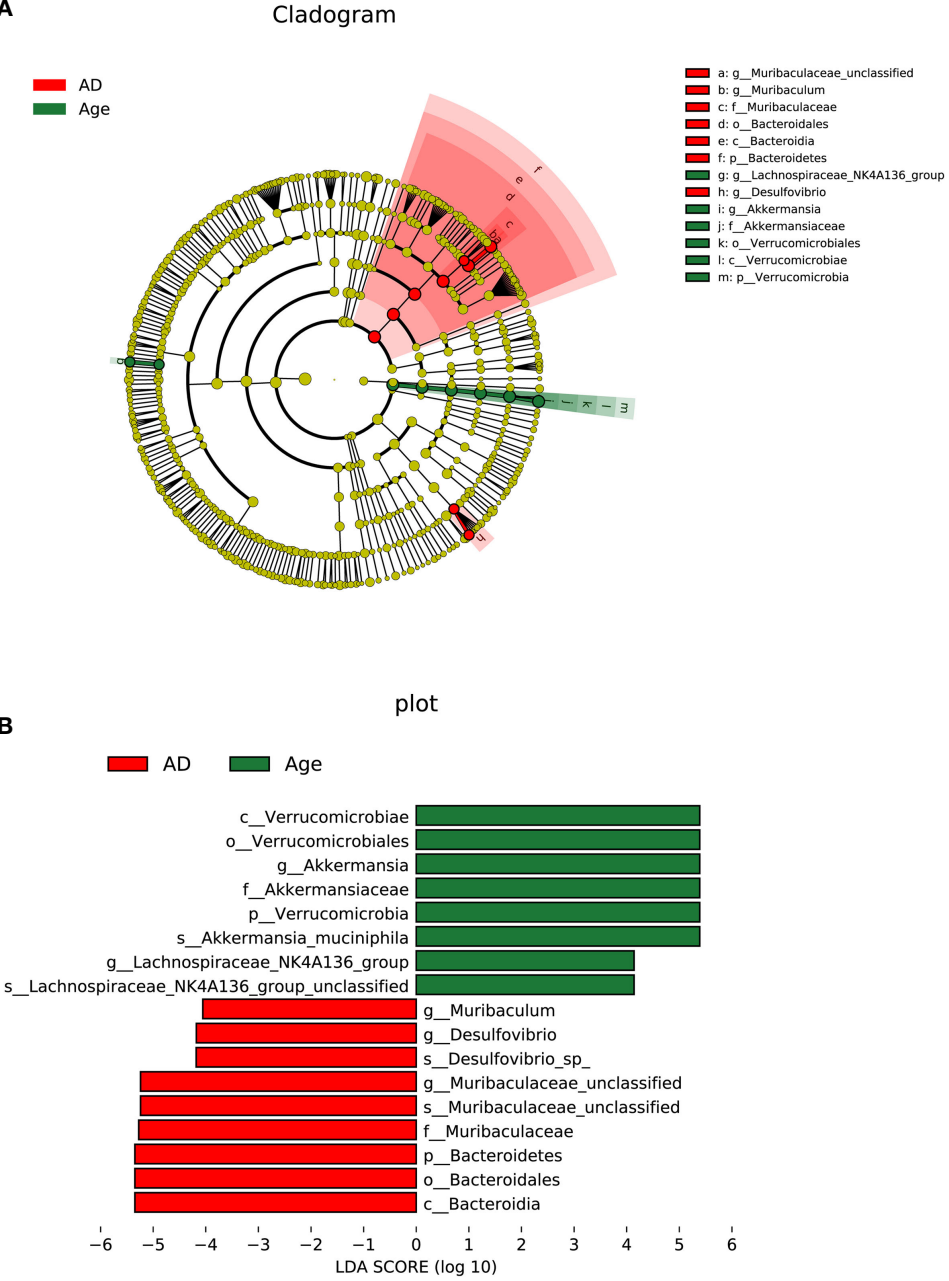
Linear discriminant analysis (LDA) integrated with effect size (LEfSe). **(A)** The circle radiating from inside to outside represents the taxonomic level from phylum to genus, and the species with no significant difference are yellow. **(B)** LDA value distribution histogram.

To investigate the microbial changes between the AD group and the Age group, we used LEfSe (LDA Effect Size) to analysis. The cladogram showed that the taxa of the Age group differed the most from the AD groups. We set the linear discriminant analysis (LDA) threshold to 4 and to explore microbial species with significant differences between the AD and Age groups. A total of 13 enriched species were identified. Among them, 7 species were enriched in the AD group and 6 species were enriched in the aged group (**Figure 5**). The results showed that at the species level, Lachnospiraceae NK4A136 group unclassified and Akkermania muciniphila were significantly enriched in Age group, and Desulfovibrio sp and Muribaculeae unclassified were significantly enriched in AD group.

### Function prediction of microbiota

**Figure 6:**
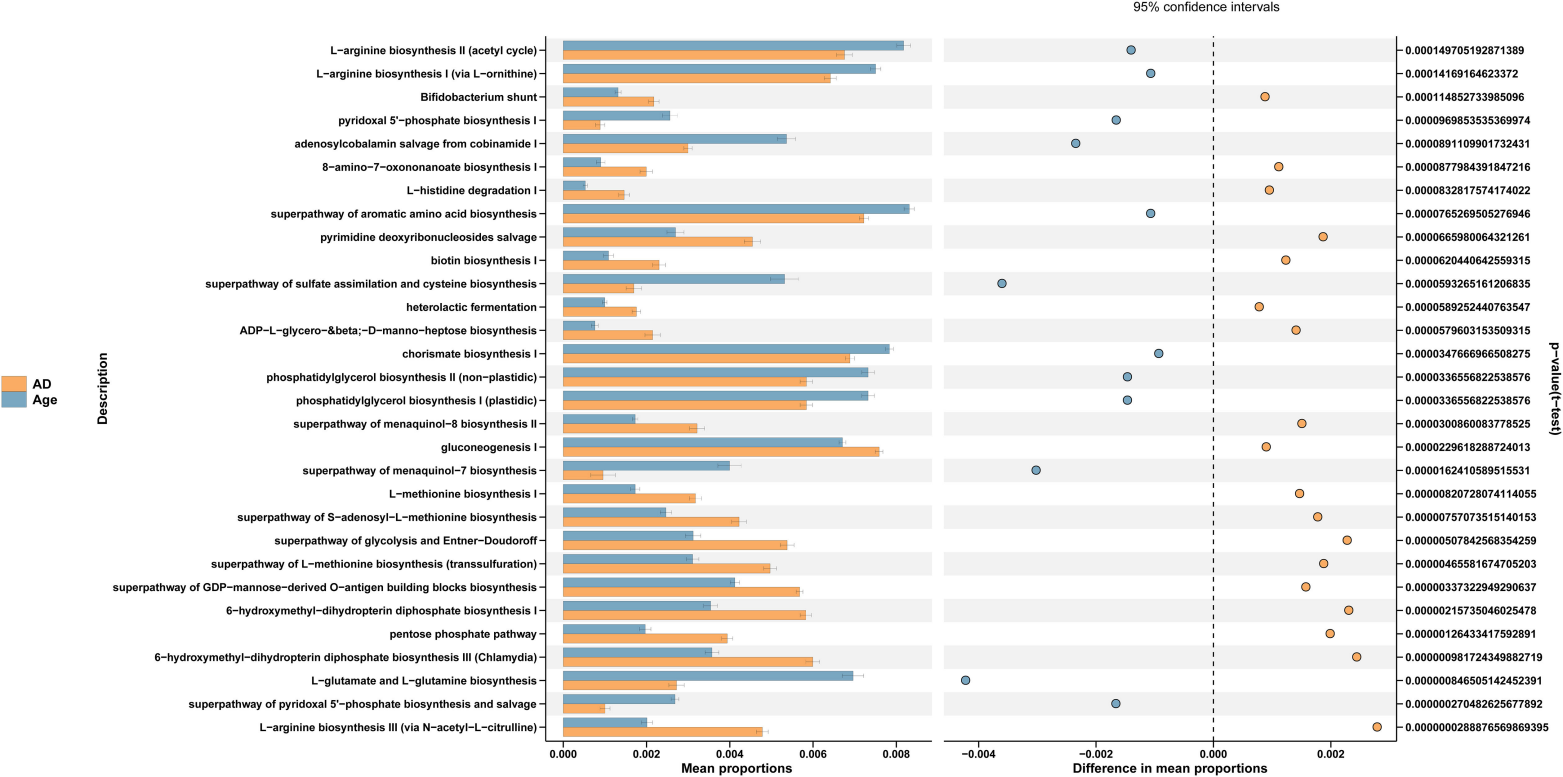
PICRUSt analysis. Functional prediction pathways in microbial differences among the groups.

We used PICRUSt to link the microbiota to different functions. The differences of functional annotation results among the groups were compared by PICRUSt analysis (**Figure 6**). The picture shows the threshold value of P value <0.05 according to t-test difference test. The functions with statistically significant differences in abundance data in the functional database are shown in the results (95% confidence interval) (**Table 1**). As illustrated in the figure, compared with the Age group, the microbiota function of the AD group was primarily related to PWY-6147, PWY-7539, PWY-6519, PWY0-1241, P124-PWY, BIOTIN-BIOSYNTHESIS-PWY, GLUCONEO-PWY, P122-PWY, PWY-5154, HISDEG-PWY, HOMOSER-METSYN-PWY, PENTOSE-P-PWY, PWY-7199, PWY-7323, GLYCOLYSIS-E-D, PWY-5347, PWY-6263 and MET-SAM-PWY pathways. They Participate in carbohydrate, amino acid, protein and other metabolic pathways of the body. In conclusion, the aged mice exhibited not only changes in the dominant microbiota and reduced relative abundance of beneficial microbiota but also changes in the metabolic pathways of the microbiota.

**Table 1:** The functional pathway.

Pathway	Description
PWY-6147	6-hydroxymethyl-dihydropterin diphosphate biosynthesis I
PWY-7539	6-hydroxymethyl-dihydropterin diphosphate biosynthesis III (Chlamydia)
PWY-6519	8-amino-7-oxononanoate biosynthesis I
PWY0-1241	ADP-L-glycero-&beta;-D-manno-heptose biosynthesis
P124-PWY	Bifidobacterium shunt
BIOTIN-BIOSYNTHESIS-PWY	biotin biosynthesis I
GLUCONEO-PWY	gluconeogenesis I
P122-PWY	heterolactic fermentation
PWY-5154	L-arginine biosynthesis III (via N-acetyl-L-citrulline)
HISDEG-PWY	L-histidine degradation I
HOMOSER-METSYN-PWY	L-methionine biosynthesis I
PENTOSE-P-PWY	pentose phosphate pathway
PWY-7199	pyrimidine deoxyribonucleosides salvage
PWY-7323	superpathway of GDP-mannose-derived O-antigen building blocks biosynthesis
GLYCOLYSIS-E-D	superpathway of glycolysis and Entner-Doudoroff
PWY-5347	superpathway of L-methionine biosynthesis (transsulfuration)
PWY-6263	superpathway of menaquinol-8 biosynthesis II
MET-SAM-PWY	superpathway of S-adenosyl-L-methionine biosynthesis
COBALSYN-PWY	adenosylcobalamin salvage from cobinamide I
ARO-PWY	chorismate biosynthesis I
ARGSYN-PWY	L-arginine biosynthesis I (via L-ornithine)
ARGSYNBSUB-PWY	L-arginine biosynthesis II (acetyl cycle)
PWY-5505	L-glutamate and L-glutamine biosynthesis
PWY4FS-7	phosphatidylglycerol biosynthesis I (plastidic)
PWY4FS-8	phosphatidylglycerol biosynthesis II (non-plastidic)
PYRIDOXSYN-PWY	pyridoxal 5'-phosphate biosynthesis I
COMPLETE-ARO-PWY	superpathway of aromatic amino acid biosynthesis
PWY-5840	superpathway of menaquinol-7 biosynthesis
PWY0-845	superpathway of pyridoxal 5'-phosphate biosynthesis and salvage
SULFATE-CYS-PWY	superpathway of sulfate assimilation and cysteine biosynthesis

## Discussion

This article discusses the microbiome differences and the changes in inflammatory indexes between aged and adult mice during sepsis. It provides a theoretical basis for studying the effect of aging on the intestinal flora of sepsis

Our results show the following: 1. The mortality of old mice is higher than that of adult mice. 2. We analysed the levels of inflammatory factors in the liver and colon of the AD group and Age group. The results suggested substantial changes in the levels of inflammatory factors in the two groups. 3. Compared with the AD group, the intestinal microbiota of the aged group showed a downward trend, the structure of the dominant microbiota changed, and the microbiota related to inflammation increased. 4. Due to the change in the dominant microbiota structure, the functional pathways of the microbiota of the aged mice with sepsis also changed.

The intestine is a complex microbial ecosystem, and its microbiome is strongly linked to human health. The gastrointestinal tract is crucial in the pathophysiology of sepsis. Studies have found that intestinal microbiota and its metabolites play an important role in a variety of diseases, and the occurrence and development of sepsis has also been proved to be related to intestinal microbiota (Deng et al., 2021; Hu et al., 2022). The imbalance of intestinal flora will breakdown intestinal barrier function and induce mucosal immune dysfunction (Wang et al., 2019). Sepsis leads to the destruction of intestinal barrier function and flora displacement, which aggravate the level of tissue inflammation and lead to organ damage (Sun et al., 2022). We compared the levels of inflammatory factors in the liver and colon between the AD and Age groups in this study. The results revealed that inflammatory factor levels were higher in the Age group. The explanation for the high acute inflammation in elderly patients includes that the low clearance efficiency of pathogens leads to the prolongation of the stimulation time of immune response, the increased susceptibility to inflammation during immune aging, and the limited physiological reserves, leading to greater feedback on the release of pro-inflammatory cytokines (Kale and Yende, 2011; Leentjens et al., 2013; Ginde et al., 2014). This may be related to the weakening of intestinal barrier function caused by ageing (Fransen et al., 2017; Fulop et al., 2017; Lin et al., 2019). The weakening of the intestinal barrier caused by ageing may lead to systemic invasion of inflammatory microbiota components. This may be connected to the poor prognosis and high mortality among elderly sepsis patients.

Comparing the intestinal microbiota of the AD group and the Age group, we found that the diversity of the intestinal microbiota decreased significantly in the Age group. Research shows that the microbial diversity of the elderly usually decreases (Woodmansey, 2007). On the other hand, the decrease in intestinal microbiota abundance may be related to sepsis. According to certain research, sepsis can induce an imbalance in the intestinal, decrease microbiota abundance and increase opportunistic pathogenic bacteria (Alverdy and Krezalek, 2017; Fay et al., 2017). In this study, the changes of intestinal microbiota in sepsis mice caused by age were mainly compared. Moreover, there were significant differences in the intestinal microbial composition between the AD group and Age group. We found that *Bacteroidetes* decreased significantly in the Age group, while *Verrucomicrobiota* had a greater advantage in the Age group. Woodmansey EJ’s study also showed that the diversity and number of *Bacteroidetes* decreased significantly in the intestinal flora of elderly individuals, (Woodmansey, 2007). *Verrucomicrobiota* was increased in elderly patients with Parkinson’s disease, and the abundance of *Verrucomicrobiota* was correlated with plasma IFN γ with a moderate correlation between concentrations (Lin et al., 2019). At the genus level, *Akkermansia* and *Clostridioides* increased significantly in the Age group when compared with the AD group, while *Ruminiclostridium, Prevotellaceae_UCG-001, Rikenella, Parabacteroides, Oscillibacter, Odoribacter, Muribaculum, Lachnoclostridium, Intestinimonas, Faecalibaculum, Anaerotruncus* and *Alloprevotella* decreased significantly in the Age group. *Akkermansia* is the only genus of *Verrucomicrobiota* found in gastrointestinal samples, and in many studies, *Akkermansia* bacteria have been shown to improve host function, regulate immunity, reduce inflammation and improve diseases (Geerlings et al., 2018; Zhai et al., 2019; Zhang et al., 2019). Interestingly, our results showed that *Akkermansia* increased significantly in the Age group and was the dominant flora in the Age mice with sepsis. Mucin is the energy source of mucin-degrading bacteria such as *Akkermansia (* Crouch et al., 2020). The main barrier against intestinal pathogens is the mucous barrier of the colon. The intestinal microbiota relies on mucus glycoproteins secreted by the host as a source of nutrition, resulting in the erosion of the colonic mucus barrier (McGuckin et al., 2011; Johansson et al., 2013; Crouch et al., 2020). *Akkermansia* is a beneficial bacterium, but too much *Akkermansia* may be associated with depleted mucus protein, resulting in the destruction of the intestinal mucosal barrier. The destruction of the mucus barrier may be related to the high inflammation of sepsis in elderly individuals. *Clostridioides difficile* can cause severe diarrhoea (Sandhu and McBride, 2018), and increase susceptibility to sepsis and lead to the loss of normal intestinal microbiome structure and function (Shreiner et al., 2015).

Studies have shown that the intestinal flora and its metabolites can decompose and metabolize polysaccharides to produce SCFAs. SCFAs could maintain intestinal barrier function, which is important for intestinal homeostasis. SCFAs not only maintain the cell barrier but also prevent the translocation of LPS from the intestinal barrier (Kelly et al., 2015; Zhou et al., 2017). Butyrate could improve the barrier function of intestinal epithelial cells by regulating IL-10, occludin, zonulin and claudins (Wang et al., 2012; Zheng et al., 2017). *Ruminiclostridium, Prevotellaceae_UCG-001, Rikenella, Parabacteroides, Oscillibacter, Odoribacter, Muribaculum, Lachnoclostridium, Intestinimonas, Faecalibaculum, Anaerotruncus* and *Alloprevotella* have been proven to be related to the formation of SCFAs (Bui et al., 2016; Koh et al., 2016; Shi et al., 2017; Van den Abbeele et al., 2020; Yao et al., 2020; Nogal et al., 2021). *Ruminiclostridium* degrades polysaccharides by producing multienzyme complexes (including xylan endonuclease and acetylxylan esterase) to produce SCFAs, such as acetate and butyrate, that can inhibit the inflammatory response (Wang et al., 2018; Xiao et al., 2020). Furthermore, *Odoribacter* and *Rikenella* are effective anti-diarrhoea probiotics that are negatively correlated with the diarrhoea index (Xu et al., 2020). *Prevotella* and *Muribaculaceae* have positive effects on the intestine *via* immune regulation and intestinal homeostasis regulation (Zhang et al., 2015; de la Cuesta-Zuluaga et al., 2017). *Parabacteroides* can reduce tumour occurrence and reduce the level of inflammatory markers in mice (Koh et al., 2020). The above conclusions show that the flora related to the formation of SCFAs in elderly individuals with sepsis is significantly reduced.

In terms of functional prediction, the metabolic pathways of AD group and the Age group were significantly different. The functional pathways related to energy generation, antioxidation and amino acid production were relatively reduced in the aged group. For example, AD group promoted L-arginine biosynthesis. L-arginine is catabolized by various enzymes to finally produce urea, proline, glutamate, polyamines, nitric oxide, creatine or agmatine (Wu and Morris, 1998). Agmatine has anti diabetes effect on diabetic animals. Agmatine can not only increase **β**-Insulin secretion of pancreatic cells but can also inhibit hyperglycemia and reduce insulin resistance in rats (Zhang et al., 2021). In addition, a systematic review and meta-analysis showed that L-arginine could significantly reduce triglyceride (TG) levels (Sepandi et al., 2019). Bifidobacterium shunt is a special pathway involving phosphoketolase activity. Hexose, such as glucose or fructose, is metabolized into acetate and lactate through this pathway and used for energy production (Lee and O'Sullivan, 2010; De Vuyst et al., 2014). The pentose phosphate pathway (PPP) is required for ribonucleotide synthesis and is a major source of NADPH. NADPH plays a key role in the redox state of cells and is also required for fatty acid synthesis (Patra and Hay, 2014). PPP generates NADPH, which is critical for the reduction of oxidized glutathione to GSH, thereby maintaining the redox homeostasis of cells (Blacker and Duchen, 2016; Xiao et al., 2018). In addition, PPP metabolic pathway can regulate the phenotype, function and survival of inflammatory macrophages (Ma et al., 2020).

Our study has some limitations. First, the sample size of the experimental groups was very small, and the results of this study need to be confirmed by other similar studies. Second, we only counted mortality after 24 hours and did not count longer-term mortality. Third, our study only explored the difference of intestinal microbiota between adult sepsis mice and aged sepsis mice, and we did not include a control group before modeling, which weakens the rigor of our study. In addition, the analysis of fecal transplantation experiments are important for future studies exploring the impact of the intestinal microbiota in the host. Regarding *Akkermansia*, other studies have reported that the abundance of *Akkermansia* decreases in elderly individuals (Collado et al., 2007; Biagi et al., 2010), so the relationship between *Akkermansia* and elderly individuals needs to be studied further and discussed.

## Conclusion

In conclusion, this study explored the differences in intestinal microbiota diversity and dominant flora structure between aged and adult mice with sepsis and it provided theoretical basis for studying the effect of aging on the intestinal flora of sepsis.

## Data availability statement

The data presented in the study are deposited in the NCBI repository, accession number PRJNA912904.

## Ethics statement

The animal study was reviewed and approved by the Life Science Ethics Review Committee of Zhengzhou University.

## Author contributions

All authors contributed to the article and approved the submitted version.

## Supplementary material

Supplementary Figure 1 Expression of inflammatory factors in liver tissue. (*P< 0.05, **P< 0.01, ***P< 0.001)

Supplementary Figure 2 Species richness. **(A)** Venn diagram between the two groups. **(B)** Shannon–Wiener index: The abscissa is the number of randomly selected sequences, and the ordinate is the Shannon index of species diversity. The saturation of the curve proves that the sequencing results were sufficient. The results show that the curve tends to be flat, indicating that the amount of sequencing data is sufficient. **(C)** The smoother the decline of the curve, the more uniform the distribution of species.

Supplementary Figure 3 Relative abundance of intestinal flora. Abundance of specific intestinal microbiota in two groups. **P*<0.05.

Supplementary Figure 4 The correlation between microbiota and inflammatory factors. **(A)** colon tissue **(B)** liver tissue.
