# Supplementary material for: Early intestinal microbiota changes in aged and adult mice with sepsis
Source: Front Cell Infect Microbiol. 2022 Dec 27;12:1061444. doi: 10.3389/fcimb.2022.1061444 (PMC9831679; doi:10.3389/fcimb.2022.1061444)
Supplement: Supplementary file 1 [file Table_1.docx]

Table 1. 24 h mortality of animals in each group

| Group | Fatality rate (deaths/total) |
| --- | --- |
| AD group | 47% (7/15) |
| Age group | 67% (10/15) |
