# Supplementary figures and images for: Early intestinal microbiota changes in aged and adult mice with sepsis
Source: Front Cell Infect Microbiol. 2022 Dec 27;12:1061444. doi: 10.3389/fcimb.2022.1061444 (PMC9831679; doi:10.3389/fcimb.2022.1061444)

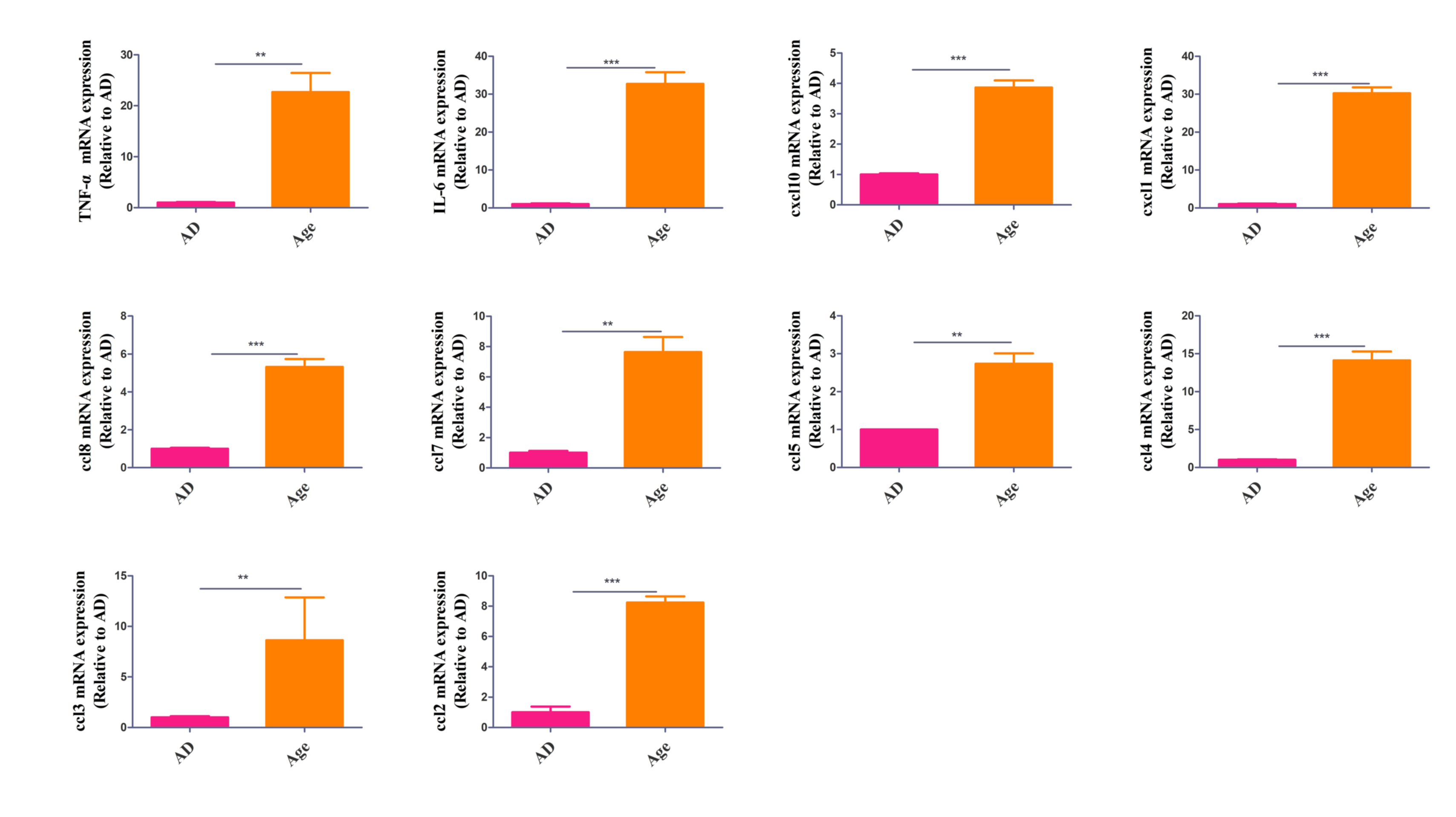

Supplement: Supplementary Figure 1 — Expression of inflammatory factors in liver tissue. (*P< 0.05, **P< 0.01, ***P< 0.001) [file Image_1.tif]

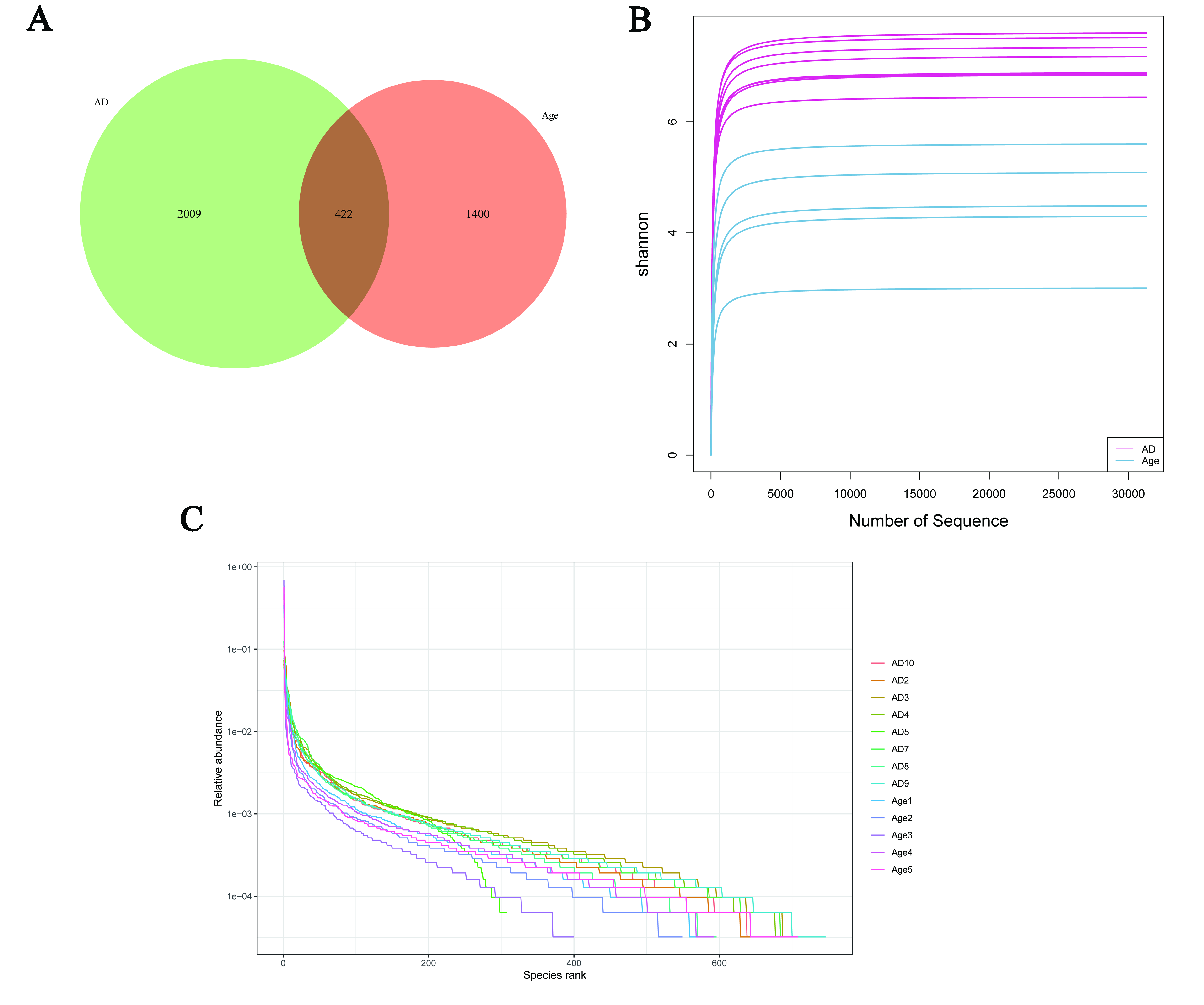

Supplement: Supplementary Figure 2 — Species richness. (A) Venn diagram between the two groups. (B) Shannon–Wiener index: The abscissa is the number of randomly selected sequences, and the ordinate is the Shannon index of species diversity. The saturation of the curve proves that the sequencing results were sufficient. The results show that the curve tends to be flat, indicating that the amount of sequencing data is sufficient. (C) The smoother the decline of the curve, the more uniform the distribution of species. [file Image_2.tif]

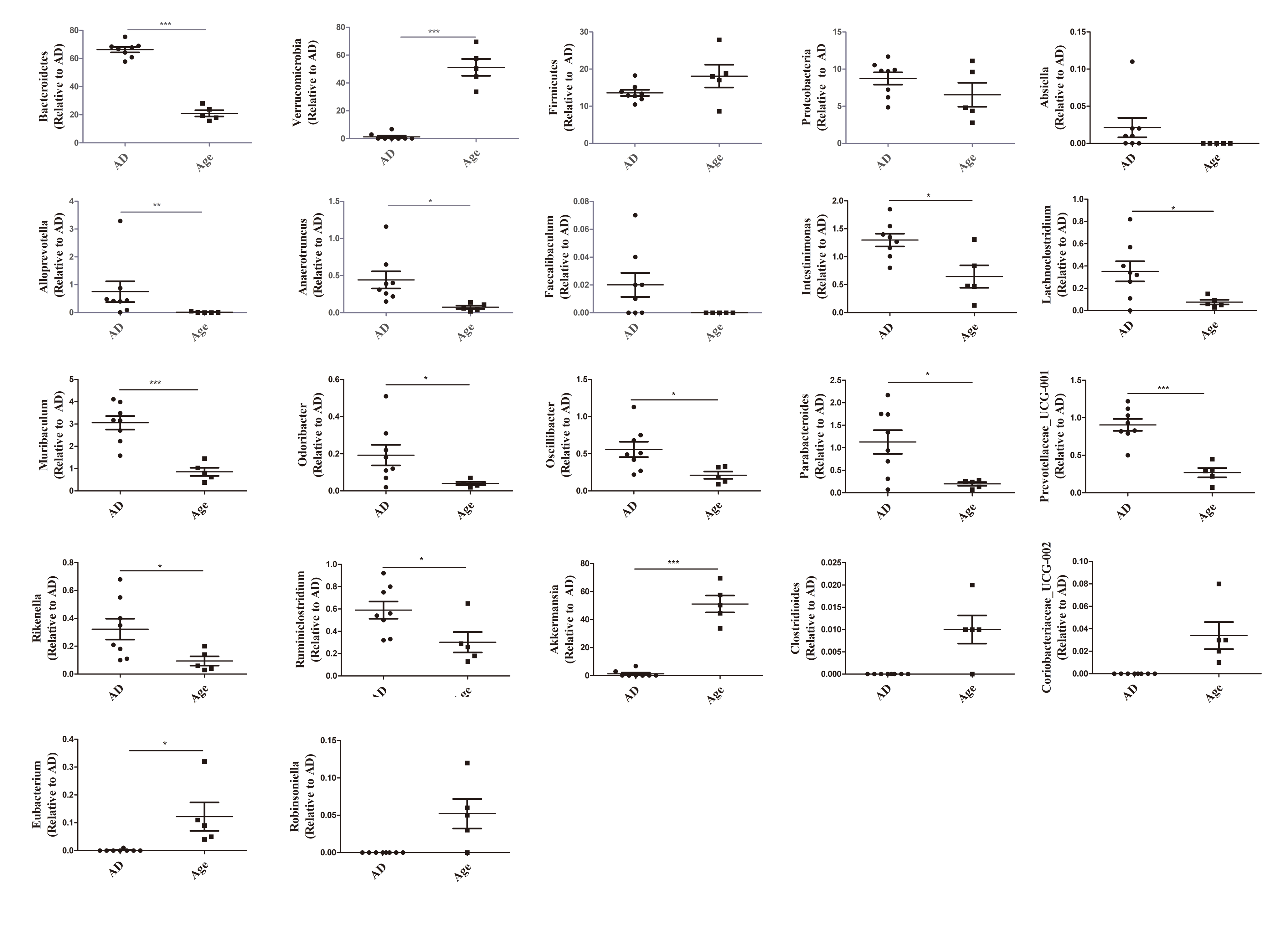

Supplement: Supplementary Figure 3 — Relative abundance of intestinal flora. Abundance of specific intestinal microbiota in two groups. *P<0.05. [file Image_3.tif]

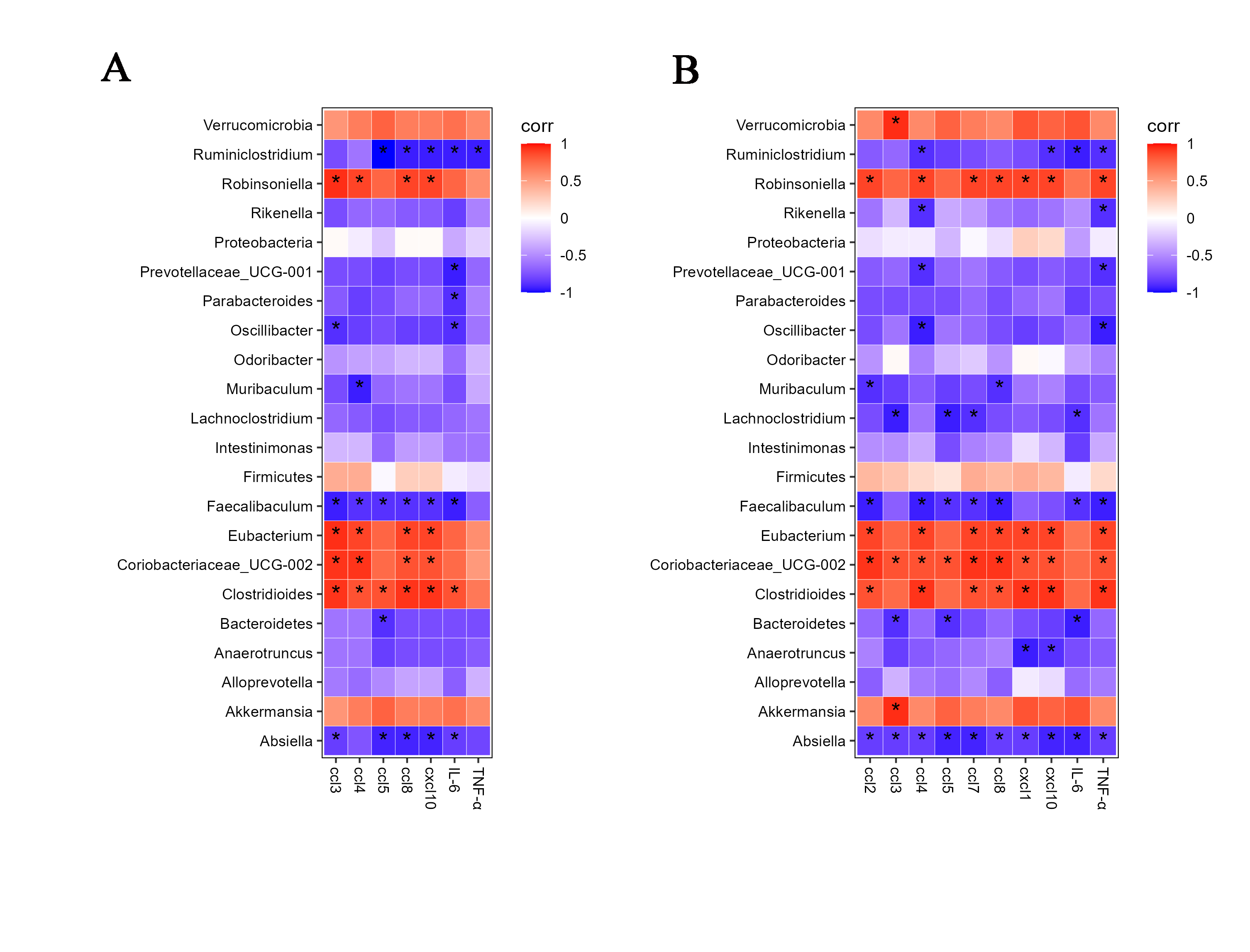

Supplement: Supplementary Figure 4 — The correlation between microbiota and inflammatory factors. (A) colon tissue (B) liver tissue. [file Image_4.tif]
